# Critical Hours and Important Environments: Relationships between Afterschool Physical Activity and the Physical Environment Using GPS, GIS and Accelerometers in 10–12-Year-Old Children

**DOI:** 10.3390/ijerph16173116

**Published:** 2019-08-27

**Authors:** Teun Remmers, Carel Thijs, Dick Ettema, Sanne de Vries, Menno Slingerland, Stef Kremers

**Affiliations:** 1 School of Sport Studies, Fontys University of Applied Sciences, 5644 HZ Eindhoven, The Netherlands; 2 CAPHRI Care and Public Health Research Institute, Department of Epidemiology, Maastricht University (Medical Center+), 6200 MD Maastricht, The Netherlands; 3 Department of Human Geography and Planning, Utrecht University, 3584 CS Utrecht, The Netherlands; 4 Research Group Healthy Lifestyle in a Supporting Environment, The Hague University of Applied Sciences, 2533 SR The Hague, The Netherlands; 5 NUTRIM School for Nutrition and Translational Research in Metabolism, Department of Health Promotion, Maastricht University (Medical Center+), 6200 MD Maastricht, The Netherlands

**Keywords:** children, physical activity, accelerometer, GPS, spatial behavior, context-specific

## Abstract

Introduction: The objective of this study was to assess relationships between children’s physical environment and afterschool leisure time physical activity (PA) and active transport. Methods: Children aged 10–12 years participated in a 7-day accelerometer and Global Positioning Systems (GPS) protocol. Afterschool leisure time PA and active transport were identified based on location- and speed-algorithms based on accelerometer, GPS and Geospatial Information Systems (GIS) data. We operationalized children’s exposure to the environment by combining home, school and the daily transport environment in individualized daily activity-spaces. Results: In total, 255 children from 20 Dutch primary schools from suburban areas provided valid data. This study showed that greenspaces and smaller distances from the children’s home to school were associated with afterschool leisure time PA and walking. Greater distances between home and school, as well as pedestrian infrastructure were associated with increased cycling. Conclusion: We demonstrated associations between environments and afterschool PA within several behavioral contexts. Future studies are encouraged to target specific behavioral domains and to develop natural experiments based on interactions between several types of the environment, child characteristics and potential socio-cognitive processes.

## 1. Introduction

Physical activity (PA) is associated with numerous health benefits in school-aged youth [1,2]. Results from observational studies generally show dose–response relationships; that is, any increment in PA, irrespective of the type, frequency or duration, is related to increasing cardiovascular, musculoskeletal, cognitive and metabolic benefits for children’s general health and well-being [2,3,4,5]. In addition, experimental studies have shown that PA programs can achieve promising results on the anthropometrical and cardiovascular risk profiles in high-risk youth [6]. As physical inactivity tends to track from youth to adulthood [7], promoting PA of children is a crucial component of strategies that combat the associated health consequences of physically inactive lifestyles.

The development of objective measurements (e.g., accelerometry) allows researchers to continuously monitor children’s daily PA behavior, and to investigate separate time periods that are promising for interventions by filtering data outputs based on time segments. Acknowledgement of these specific time segments is essential, as children’s PA patterns fluctuate during the day, and can be highly context-specific [8,9]. The afterschool period is such a context-specific time segment that is often referred to as “critical hours” for PA promotion, because it contributes up to half of the daily amount of moderate to vigorous PA (MVPA) [10], and afterschool PA declines as children reach adolescence [11]. During the afterschool period, children have more discretion over the activities in which they engage [11,12]. Because of this, afterschool behaviors may reflect more autonomously regulated behavior, which may be more likely to persist into habits and routines [13,14]. Hence, afterschool PA has been considered highly predictive of overall sustained PA patterns [15], which makes it a primary time segment for PA interventions [16].

Since 2010, the number of studies that used objective measurements of PA to investigate afterschool PA rapidly increased [17]. However, interpretation of results of the studies to date is limited by several challenges. First, there are inconsistent definitions of the afterschool time segment [11]. For example, while some studies used generic start time thresholds such as 3 PM [12,18,19,20] or 3:30 PM [21,22,23], other studies used reported schools’ schedules [10,11,24,25,26,27] or excluded activity during school hours [28]. Second, during the afterschool period children are exposed to diverse attributes of the physical environment which may have an influence on children’s PA [29,30]. Hence, various studies have investigated attributes that foster afterschool PA. To date, six studies have investigated relationships between afterschool PA and the physical environment using objective measures of PA [22,25,28,31,32,33]. Four studies solely investigated environmental features around the residential neighborhood [22,25,31,33]. However, not only the residential environment, but also other environments such as the children’s school environment and the daily transport route between home and their school are of interest when investigating environmental attributes related to afterschool PA, as especially during the afterschool period, children spend significant parts of their time outside their residential neighborhood [34]. Consequently, the second challenge is to analyze children’s PA in relation to so-called daily activity spaces (e.g., including school, residence and daily transport route as spatial anchor points) may be especially suited for investigating associations with PA during the afterschool time period [35,36]. Third, although investigating afterschool PA separately from total daily PA is an important first step in examining context-specific determinants of PA [32], the afterschool period still consists of multiple distinct “activity types” (e.g., active transport, organized sports participation and leisure time PA) [24]. The ability to differentiate between several activity types is essential, as the influence of potential (environmental) determinants depend on these activity types [9]. Only very few studies to date, however, have succeeded to measure these activity types and attributes of the physical environment using objective measurements [30]. Thus, the third challenge is to differentiate between activity types in the afterschool time period, in order to understand which attributes of the physical environment influences specific types of objectively measured PA [9].

Combined accelerometer and Global Positioning Systems (GPS) methodologies may help to overcome the challenges presented above. First, afterschool time segments may now be individually validated by the actual presence of a child on its school parcel, rather than based on generic start-time thresholds or reported school schedules. Second, when integrated with registries of the physical environment such as Geographic Information Systems (GIS), researchers can measure underlying characteristics of the environment based on a participant’s unique GPS-based mobility pattern. In this way, children’s environmental exposure can be measured in a broader perspective than solely their residential environment, as operationalized in the majority of previous studies [29,30,37]. Third, when combining the parameters activity, location and time, combined accelerometer and GPS methodologies can provide essential information on the relative importance of afterschool activity types and the context in which these occur. This may increase our understanding of the influence of attributes of the physical environment in fostering these afterschool activity types.

To date, three studies have investigated the influence of the physical environments on afterschool PA, using accelerometers and GPS methodologies [21,24,28]. These studies have all used a so-called contemporaneous momentary design, in which accelerometer-measured activities and GPS-derived geographical location (and its attributes of the physical environment) are collected at the exact same moment in time. In other words, these designs investigate relationships between behavior and environment within simultaneously occurring pairs of (1) PA or sedentary behavior and (2) attributes of the physical environment. However, these designs may be vulnerable to selective daily mobility bias [35,37]. Selective daily mobility occurs when researchers investigate simultaneously occurring PA-environment pairs, and include locations that children purposively visit to perform the desired behavior (e.g., visiting sports grounds for performing the planned PA) [35,38,39]. In this case, individual preferences for performing PA behavior in specific settings confounds the causal relationship between environmental exposure and PA behaviors [37]. Although these studies can reveal relevant short-term insights into where PA takes place [28,40,41,42,43,44], it remains unclear whether long-term PA patterns are actually influenced by attributes of the physical environment [38,39].

One possible way to overcome potential selective daily mobility bias is to reconsider our view of environmental exposure towards more non simultaneous or long-term exposure of the environment. Based on work in space–time geography, transportation research and environmental psychology, Perchoux and colleagues conceptualized a theoretical framework of environmental exposure [35]. This theoretical framework defines environmental exposure as a dynamic individualized activity space based on space and time [45]. These individualized spaces are shaped by the location of usual visited places (e.g., home or school) [46]. Furthermore, individual spatial freedom is delimited by various constraints (e.g., transportation mode or regulations concerning home or school hours) [47]. Moreover, these individual activity spaces are also formed by the social interactions that are often fixed by location or time, and may over time even evolve to a sense of belonging to a certain neighborhood [48]. Relative accessibility of PA opportunities within a child’s daily activity space may influence its afterschool PA behavior. The aim of the present study was thus to improve our understanding of the relationship between characteristics of children’s daily physical environment and their afterschool PA behavior, by using combined accelerometer and GPS methodologies, and framing analyses within the activity space framework [35].

## 2. Materials and Methods

### 2.1. Design and Participants

The present study was based on the baseline measurement of the PHASE study, which examined longitudinal relationships between the built environment and children’s PA patterns in the transitional phase from primary to secondary schools. The PHASE study was conducted in the municipality of ‘s-Hertogenbosch (the Netherlands), which covers around 110 square kilometers, and has approximately 150,000 residents [49]. Population density varies between municipality neighborhoods (1.8–59.0 residents per hectare). Average population density of included neighborhoods was 19.4 residents per hectare. For the baseline measurement, we invited 30 primary schools to participate (initial sample ≈ 1000 10–12-year-old children), of which 20 schools agreed to participate. Children in the final year were all invited to participate in a 7-day accelerometry and GPS monitoring protocol. We recruited children by informing teachers and staff, distributing pamphlets at schools, conducting two educational presentations by the research staff at schools and by distributing letters for parents or guardians. One year later, participating children were approached again at their secondary school for a follow-up measurement using the same protocol. For the current analyses, only baseline measurements were considered. Ethical approval for the PHASE study was obtained from the research ethics committee of the Maastricht University Medical Center (reference number 12-4-077).

Baseline data were collected from April till July 2015, with an average daily temperature of 15.12 degrees Celsius (SD = 4.96) and 77% of the days with <1.0 mm of precipitation (based on registries from a local municipality weather station). Sunset times during this time period in the center of the Netherlands were between 20:13 and 22:06 h [50].

Accelerometers and GPS loggers were distributed during school hours, where children received verbal and written instruction about how to wear the devices. Both devices were attached to the waist and worn at the right hip with a single elastic belt. We instructed children to wear the belt during waking hours for 7 consecutive days, only to remove the belt during water-related activities (e.g., swimming and showering), and to recharge the GPS logger every day before going to sleep. Children were asked to record the times and reasons why they took off the devices in a diary. After measurement, devices were collected by the research staff during school hours, while the child and one of their parents received a verbal and written invitation for an electronic questionnaire.

### 2.2. Measurement

#### 2.2.1. Socio-Demographic Measures

Parents reported children’s name, school, date of birth and address, directly after providing informed consent to participate in the study. In addition, we assessed whether children from divorced parents were residing at two locations. Schools provided detailed class timetables for the data-collection period. For each participant, we computed the social economic status score of their residential neighborhood, which summarizes the average income per household and the percentages of households with low income, without a paid job and with a low education level [51]. Directly after measurement, children and parents filled in an electronic questionnaire focusing on aspects such as perceived environment, the child’s behavior and homework.

#### 2.2.2. Accelerometer Measures

Accelerometers provide reliable and accurate measures of youth’s PA patterns [52,53]. In this study, accelerometers (GT3X, ActiGraph, Pensacola, FL, USA) were set to record data at 10 s epochs. The manufacturer’s software (Actilife version 6.11.9) was used for initialization and initial screening of data output. In order to manage the data load of subsequent analyses, at least 600 min of wear time per day for at least two weekdays was applied [54].

#### 2.2.3. GPS and GIS Measures

The GPS logger used in this study (BT-Q1000XT, Qstarz International Co., Taipei, Taiwan) showed relatively good static spatial accuracy compared to other units [55], and acceptable dynamic accuracy [56]. We used the manufacturer’s software (QTravel version 1.46) for initialization and downloading data output. In order to optimize sample frequency while considering the limited data-storage capacity of the GPS when using a 7-day protocol, devices were set to record data at 10 s epochs. Furthermore, we configured the device to record date, time, longitude, latitude, elevation, speed, signal-to-noise ratio, number of satellites in reach and to stop logging when storage capacity was full [24]. GIS data was extracted at the time of measurement from the municipality of ‘s-Hertogenbosch. This means that extraction of environmental data and the collection of GPS and accelerometer data commenced at the same time period. GIS-data of this municipality consisted of two levels. In the first level, categories were vegetated terrain, water, buildings and roads. In the second level, subcategories existed for vegetated terrain and roads (e.g., woods, lawn, shrubs and agriculture, and then cycling paths, rural roads and highways for vegetated terrain roads, respectively).

### 2.3. Data Analysis

#### 2.3.1. Data Management and Validation

Accelerometer and GPS data were processed using the Personal Activity and Location Measurement System (PALMS), which allows users control over most parameter settings in a web-based application [57,58]. Intensity of accelerometer activity was categorized into sedentary behavior, light PA (LPA), moderate PA (MPA) and vigorous PA (VPA) according to Evenson’s cut-points [59], which performed best in free-living activities of 5–15-year-old children [60]. We defined non-wear time as ≥20 consecutive minutes of zero accelerometer counts [61]. PALMS processed GPS data by filtering invalid values according to extreme speed (i.e., threshold ≥ 130 km/h) and extreme changes in elevation (i.e., threshold ≥ 1000 m). We applied the same PALMS algorithms (version 4) as Carlson et al. for trip and trip mode classification (e.g., pedestrian or bicycle) [62]. The present study applied 10–25 km/h bicycling speed thresholds, while Carlson et al. applied thresholds of 10–35 km/h because their sample consisted of commuting cyclists that were expected to accumulate higher cycling speeds. Invalid GPS points were imputed from the last known valid point, for up to 10 min. Finally, we ordered PALMS to match data based on start- and end-times of the GPS logger. The PALMS dataset resulted in 10 s GPS epochs (e.g., latitude, longitude, trip mode and speed) with timestamp-merged accelerometer data (e.g., activity counts and activity intensity classification).

**Figure 1:**
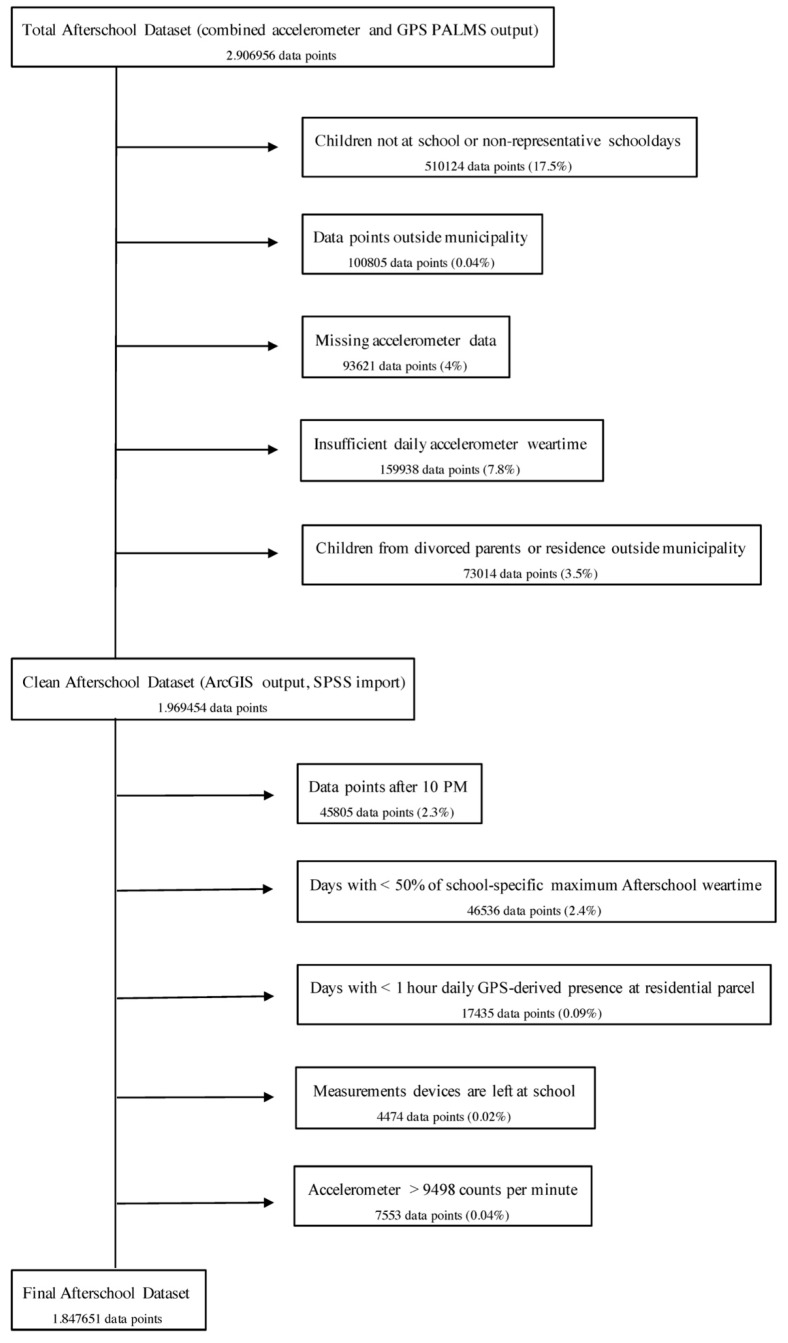
Data flowchart.

We exported PALMS datasets separately for each school and integrated these separate datasets into a PostgreSQL database (http://www.postgresql.com). We first deleted data from pre-selected participants with insufficient accelerometer wear time. Subsequently, based on reports from the school principal, we performed queries to identify relevant time segments based on an individual school’s schedules. Time-segmented datasets, containing both accelerometry and GPS data, were integrated into ArcGIS version 10.4.1 (ESRI, Redlands, CA, USA). Similarly, we overlaid GIS data from the municipality of ‘s-Hertogenbosch. We applied basic validation rules by deleting records (i.e., 10 s epoch measurements) with incidental missing accelerometer data, or records that were located outside the municipality study area. In accordance with previous studies investigating afterschool time segments [11,21,32], we ensured reliability of the time segment by only selecting records from days with >4 h of valid wear time in the afterschool time segment (see Figure 1).

#### 2.3.2. Spatial Analyses to Validate Afterschool Leisure Time PA

We identified the behavioral domains in which children participated afterschool, based on the GPS-derived context information and the hierarchical decisions of Klinker et al. [63]. First, we geo-located each participant’s residential location, school buildings and the accompanied geo-referenced parcels. Second, we identified the subdomain “home” by selecting records that were within 10 m of each respondent’s self-reported residential parcel from all GPS points in the afterschool time segment. For transport, we applied the above-described PALMS speed thresholds. Third, we identified four more subdomains by identifying records within 10 m from the following geo-referenced parcels: School, sports facilities, shopping centers or malls, or afterschool childcare. Specific parcels were identified from the municipality GIS registry. All other non-selected records were identified and hereafter defined as afterschool “leisure time” records (i.e., behavior not in transport, not at home or at school, and not at sports facilities, childcare or shopping centers).

Subsequently, we validated children’s daily presence at the school ground during school hours by calculating the daily percentage of GPS points that were on the school’s geographic parcel during the last hour of school time, while acknowledging a 10 m threshold to account for potential imprecision of the GPS logger [24,64,65]. Time segments from children with <80% of their GPS points within the school parcel during this last hour of school time, were carefully inspected in individual ArcGIS maps (see Figure 1).

#### 2.3.3. Spatial Analyses of Children’s Daily Activity Spaces and GIS Data

**Figure 2:**
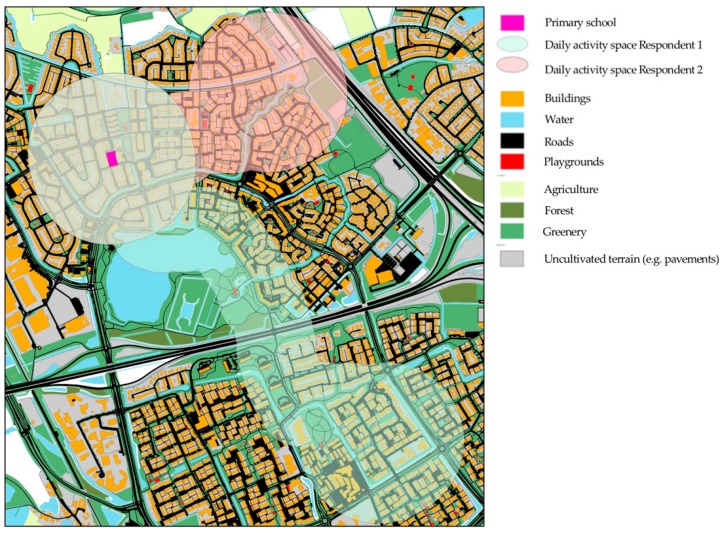
Example of children’s activity spaces and accompanied visualization of GIS data.

We computed a 400 m Euclidean buffer surrounding each participant’s school and residence. This buffer size is in line with other studies in terms of the age group of participants and relative density of facilities in the environment [32,66,67,68,69]. We also computed a Euclidean buffer surrounding the shortest path (based on the combined cycling paths and street network) between participant’s school and residence along the street network. For this daily transport route, we argued that a 200 m buffer would be appropriate given the relatively high density of streets, buildings and PA facilities, and the short distance between school and home in this specific population. Subsequently these three buffers (i.e., home, school and daily transport route between home and school) were combined into an individualized daily activity space. Relevant GIS-data from the municipality of ‘s-Hertogenbosch was extracted from these daily activity spaces, using the spatial join and summarize functions in ArcGIS (see Figure 2). For each respondent, we first computed the total area (in square meters) of their daily activity space. Subsequently, for each daily activity space, we computed the proportion of various environmental attributes relative to its daily activity space. For example, a participant’s relative accessibility to cycling paths was quantified as the area (in square meters) of cycling paths within its each participant’s individual activity space, relative to the participant’s own daily activity space.

### 2.4. Statistical Analyses

Days were used as the unit of analysis because this allows examining day-to-day variation within children. After presenting participant characteristics (Table 1), we present the median and interquartile ranges (IQR) for children’s daily contribution to several domains and subdomains (Table 2) [63]. In our multivariate analyses, we focused on the leisure time PA and the active transport domain. In the leisure time domain, associations were separately analyzed for LPA and MVPA. Active transport (i.e., cycling and walking) were identified by GPS-determined speed thresholds and were analyzed as one combined intensity category. The first set of independent variables were meteorological variables, accessed from a local municipality weather station’s registry on an hourly basis. The second set concerned independent variables from the daily activity spaces, in which we focused on public environmental features accessed from the municipality’s GIS registry.

Comparable with the multilevel statistical approach of previous studies [24,62,70], we accounted for the potential clustering of children within schools using a random intercept, based on moderate clustering especially at higher intensities (ICC = 0.01 and 0.07, for light PA and MVPA, respectively). We also accounted for the clustering of days within children by assigning a random intercept and slope for days within children (based on ICC = 0.12 and 0.09, for light PA and MVPA, respectively).

Normality of residuals were inspected using normal probability plots. As all of our model-residuals showed significant deviation from normality, we transformed our dependent variables using log-transformations, while adding a constant of 1 to all values. Subsequently, the model fit of these models were inspected and tested against the non-transformed variant to verify its fitting capabilities. To facilitate comparisons, we standardized our independent variables in multivariate models.

In analyses containing second-level GIS-features of the built-environment, a manually executed stepwise procedure was followed, in which potential multicollinearity issues were identified and controlled. Namely, based on the two-level structure of our GIS-data, first we investigated associations between our dependent variables and all first-level GIS variables, adjusting for baseline variables (i.e., wear time, age (in years), gender (boys vs. girls), temperature (mean degrees Celsius), atmospheric pressure (mean hPa), rain (0.2–10.0 mm vs. no rain), wind (mean km/h), solar exposure (UV index) and socio-economic standardized “status score” of the neighborhood compared to the national Dutch average; see Table 3).

Second, we replaced the two first-level GIS variables that contained GIS subcategories (vegetated terrain and roads) with their accompanied subcategories (i.e., second-level GIS variables). We tested these associations independent from each other. Third, we simultaneously entered second-level variables with strong associations (*p* < 0.10), and deleted variables with the largest *p*-value; only retaining variables that were statistically significant (*p* < 0.05). Baseline variables were not deleted from these models. Sensitivity analyses were also performed for potential gender differences by inspecting interaction terms, but significant interactions were not found. Likewise, we also investigated moderation for the daily minutes that respondents spent in their activity space (based on their GPS location), but no such moderation was found. Statistical analyses were performed using SPSS 21.0 for Windows (IBM SPSS Inc., Armonk, NY), and *p* < 0.05 indicated statistical significance.

## 3. Results

### 3.1. Participant Characteristics

In total, 117 boys and 138 girls from 20 primary schools provided valid data. Children were approximately 12 years old. After data-cleaning and applying exclusion criteria, 808 valid days of measurement were retained and 74% of the children provided valid data for at least three days. This resulted in a dataset of around 1.8 million records (i.e., 10 s epochs), which hold around 5000 h of afterschool data collection. Results from an electronic questionnaire that was administered after the measurements (response rates of 77% and 75% for the children and parents, respectively), shows 92% of the parents reported that their child used active transport to travel to and from school. In addition, 77% reported that their child spent equal or less than 10 min per day on afterschool homework (Table 1). On average, children lived 696.1 m (SD = 735.3) from their school, resulting in an average activity space of 1.1 (SD = 0.41) squared kilometers. Based on the respondent’s postcode area, we derived a standardized social economic status score of neighborhoods [71]. The average standardized score (mean = 0.06, SD = 1.31) shows that neighborhoods of our participants are comparable with the general Dutch neighborhoods, with considerable variability between neighborhoods. Descriptive statistics of all variables analyzed subsequently are presented in the Table A1.

**Figure 3:**
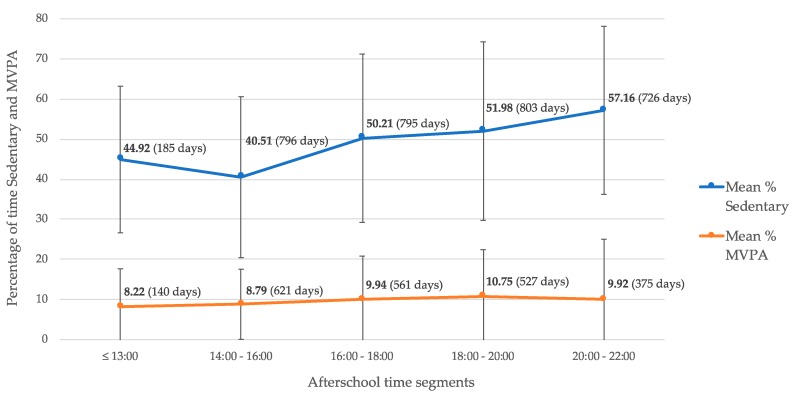
Children’s percentage of time spent sedentary and in moderate to vigorous physical activity (MVPA) across five afterschool time segments.

On average, higher percentages of sedentary time were found at later time segments (Figure 3). Similarly, MVPA mean percentages were also slightly higher at later time segments. The mean percentage MVPA between 8 and 10 PM was based on only 375 days (46%) from the total measured days. This means that the remaining 433 days (54%) contained no MVPA between 8 and 10 PM.

### 3.2. Participant’s Afterschool Behavior in Various Contexts

On average, children were most active on sports grounds (41 min, 41% from total recorded minutes in sports grounds) and during active transport (15 min, 37% from total minutes during active transport). Most of the children participated in these activities at least once in the measurement period. Children were least active on their residential parcel (8.3 min, 4% from total minutes recorded on their residential parcel). During afterschool leisure time, 22.3 min (13% from total minutes spent in that domain) were moderate to vigorously active (Table 2). Boys spent significantly more average MVPA minutes at school grounds, mean = 2.8 (SD = 4.9) versus 0.9 (SD = 0.1), and at sports grounds, mean = 13.8 (SD = 1.3) versus 7.7 (SD = 0.9). Girls spent significantly more MVPA time at their residential parcel, mean = 3.7 (SD = 0.4) versus 2.8 (SD = 0.2), and spent more time in light PA during leisure time, mean = 27.5 (SD = 1,1) versus 23.8 (SD = 1.0).

We also investigated the amount of time that children spent in their activity space (i.e., within the school, residence and daily transport buffer; Figure 2). This percentage varied across the afterschool behavioral contexts. For example, we found that in 68% of the monitored days, children spent at least 80% of their total afterschool leisure time within their own activity space. In addition, in 63.3% of the monitored days, children spent at least 80% of their total time spent in active transport within their activity space. This means that the vast majority of children’s afterschool leisure time and active transport behavior actually occurred within their own activity space as delineated by the combined home-, school-, and daily transport-route buffer.

### 3.3. Meteorological Circumstances

Baseline data were collected from April till July 2015, with an average daily temperature of 15.12 degrees Celsius (SD = 4.96) and 77% of the days with <1.0 mm of precipitation. The average atmospheric pressure was 1019.1 (SD = 6.3) hPa. The average UV-index of the sun was 2.2 (SD = 1.4). Average amount of wind was 8.2 (SD = 4.2) kilometers per hour. In terms of general characteristics of the physical environment, water accounted for approximately 16.0% of the children’s activity space, roads for 29.5%, vegetation for 32.2% and buildings for 19.6%.

Meteorological circumstances significantly affected children’s leisure time PA and transport PA. Days with at least 0.2 of total rain (versus no rain) were associated with less MVPA leisure time and fewer active minutes in cycling and walking. In addition, higher temperatures were related with more active minutes while cycling and less activity in MVPA leisure time (Table 3). In subsequent models, meteorological circumstances are controlled for.

### 3.4. Association between PA and First-Level Attributes of the Physical Environment

Larger activity spaces, typical for children that lived further from their school (see Figure 2), were associated with fewer active minutes of walking afterschool, and more active minutes of cycling. We found that in afterschool active transport, a higher spatial density of buildings was related with less cycling, while longer distances from home to school were related with more cycling (Table 3). First-level environmental features were not associated with afterschool leisure time PA.

### 3.5. Association between Afterschool Leisure Time PA and Second-Level Attributes of the Physical Environment

Multivariate results (Table 4) showed that smaller distances from school to home and higher spatial densities of agriculture, lawns, shrubs and local roads were associated with more minutes of LPA. Higher density of highways was associated with less minutes of leisure time LPA. In addition, more minutes of leisure time MVPA were associated with a higher density of lawns, shrubs, pedestrian paths and smaller distances from home to school.

### 3.6. Association between Afterschool Active Transport and Attributes of the Second-Level Physical Environment

Higher densities of buildings, lawns and pedestrian paths were associated with less minutes of cycling (Table 5). More minutes of cycling were associated with a higher density of pedestrian areas. Finally, more minutes of walking were related with smaller activity spaces, a higher density of agriculture, shrubs and pedestrian paths.

## 4. Discussion

This study examined relationships between features of the physical environment and specific afterschool PA behaviors (i.e., afterschool leisure time PA behavior and afterschool active transport). Our first, more methodological aim, was to investigate context-specific afterschool leisure time and active transport by filtering these contexts from other afterschool contexts, such as organized sports participation. This may be important since previous studies suggested that this may be a confounding factor in the relationship between PA and the physical environment [37,72]. We showed that GPS devices provide additional descriptive information about the context of daily PA and mobility patterns, which enables more context-specific analyses of the relations between leisure PA and its environmental determinants [9].

### 4.1. Empirical Findings

This study showed that greenery density (i.e., lawns and shrubs) was associated with more afterschool leisure time PA and walking, but we found no association with the density of general vegetation. Systematic reviews, including studies until 2010, reported a mixed association between environmental greenspaces and PA [29,73]. However, studies from 2010 onwards using objective PA and GPS-determined environmental exposure consistently suggest that children are more active in greenspace environments such as parks [28,41,74,75]. Findings from the present study not only support the suggestion that shrubs and lawns (often found in public green spaces) may be important facilitators for children’s PA, but also show that children with a higher density of shrubs and lawns in their activity space, generally perform more afterschool leisure time PA than children with a lower density of these environments (irrespective of whether PA is actually performed around shrubs or lawns).

From the six studies that investigated relationships between objective afterschool PA and the physical environment [22,25,28,31,32,33], two studies focused on public features of the environment [25,32]. These two studies suggest that not only the public open space closest to the children’s residence is associated with afterschool PA, but also the larger home–school environment or parts thereof. In contrast to an earlier study [32], we found no evidence that a higher density of public playgrounds was associated with more afterschool leisure time PA or active transport. This may be explained by the fact that GIS data in the present study did not enable us to look at quality, maintenance status or age-appropriateness of these playgrounds. In addition, some unstructured PA at playgrounds may have been lost when excluding behavior performed at sports facilities. Additional use of activity diaries may help in identifying planned PA behavior at specific locations. Similarly, we found that a higher density of buildings was associated with more minutes of leisure time MVPA. Results may be comparable with results from Rodriguez et al., who found increased MVPA in environments with a higher population density.

We found that the distance between the children’s school and home was an important determinant of leisure time PA, cycling and walking. More specifically, greater home–school distances were related to more cycling, but less leisure time MVPA and walking. As results from the electronic questionnaire showed that the vast majority of our participants used active transport to get to and from school, greater distances may be related to more cycling as a replacement of walking during the home–school commute (and vice versa). In addition, greater distances may also reflect the subgroup of participants living in neighborhoods that may be somewhat further away from facilities (e.g., supermarkets or afterschool activities) [75,76]. This was supported by our finding that a lower density of buildings and pedestrian paths (typical for less urbanized areas) was also associated with more cycling. Moreover, we found that a higher density of main roads was associated with more cycling. As a higher density of main roads may relate to lower connectiveness for cyclists, this relationship may be explained by an increased likelihood of detour trips. For example, in order to visit the desired location, children are forced to take detours to safely cross main roads, which in turn may result in increased daily minutes of cycling. The same explanation may be valid for the unexpected positive relationship between pedestrian areas and cycling. As cycling is usually prohibited in pedestrian areas, children may be forced to take detours, which in turn may increase daily minutes of cycling.

### 4.2. Methodological Considerations

Systematic reviews investigating relationships between the environment and PA urged for objective measurements of both PA and the physical environment [29,73]. Consequently, there has been an increase in studies combining accelerometer and GPS measurements, to investigate relationships between an environment and behavior using contemporaneous momentary designs. In this design, objective data on characteristics of the environment (based on the GPS location) and PA intensity are analyzed in simultaneously occurring PA–environment pairs [28,34,41,44,74,75,77,78,79,80]. However, as PA is a complex interplay between spontaneous and planned behavior, involving memory of PA facilities, time and capacity constraints, social interactions and compensation mechanisms [81,82,83], children’s behavioral response may not occur simultaneously with exposure to PA-supportive attributes in their direct physical environment. Furthermore, studies using contemporaneous designs are vulnerable to selective daily mobility bias [35,37].

Quality and specificity of GIS data may depend from one data source to another. As variability in the quality and data structure of GIS data hampers between-study comparisons, researchers are encouraged to provide insight into the various levels of spatial detail underlying their spatial analyses. The present study’s GIS data consisted of high-quality, fine-grained polygons that were routinely validated, but presented a rather rudimentary categorization of the physical environment. Increasing the specificity of GIS-based categorization may pinpoint more precise environmental attributes, but on the other hand may be more vulnerable to erroneous classifications.

Children’s daily activity spaces aggregate to the same neighborhood environment if children live closer to school (see Figure 2). The percentage of “shared” neighborhood environment (and thus the added value of using individual activity spaces instead of regular residential or school environments) depends on the distance children reside from their school. This means that future studies are encouraged to make informed decisions about children’s daily exposure or accessibility to their environment, based on distances between school and homes, and knowledge of potential other frequently visited anchor points. For example, some studies incorporated the participant’s own perceptions of their daily mobility environments (i.e., neighborhoods) [39].

In the present study, we have reconsidered children’s environmental exposure in the afterschool time period, following the theoretical framework of Perchoux et al. We created individual daily activity spaces for each child by assigning residential and school environments, in combination with the shortest route between these locations. Multiple subjective experiences of the same attributes of the physical environment may lead to increasing knowledge about the environmental qualities within their activity space [28,35,37], and a sense of belonging to a certain neighborhood [48]. Environmental PA opportunities within a child’s “belonging neighborhood” may have more influence on PA behavior than more distant PA opportunities [32]. In addition, social networks also influence daily activity spaces [48]. For children, the residence and school are prominent locations from which these social networks may arise in the afterschool time period. Therefore, we theorized that afterschool daily mobility due to social opportunities (or constraints) may center around the children’s residence and their school. In conclusion, we propose this operationalization of environmental exposure area to investigate meaningful relationships between environmental attributes that are located within a child’s daily area of influence, and children’s afterschool PA behavior. In our view, this may be an important step forward in understanding causal mechanisms between relative accessibility or opportunity to various characteristics of the objective physical environment and afterschool PA in primary-school children.

### 4.3. Strengths and Weaknesses

Study strengths are the utilization of GPS devices in order to investigate associations between the objectively assessed built environment features (using GIS) and domain-specific PA, adjusting for meteorological differences and the nested structure of measurement days within children, and children within schools.

We however acknowledge that in some instances GPS data may not indicate the true behavioral context of the location that was visited (e.g., sports grounds visited for sports participation or as a spectator). Although it may be possible to improve validity of the GPS signal by (random) confirmation of the participant, this comes with the cost of participant burden. In addition, a small proportion of the afterschool records (0.04%) were located outside the municipality, of which we had no GIS data available. If these records may have consisted of regular PA participation, our results may have been biased. However, considering the small percentage of records outside the municipality, we expect the magnitude of this potential bias to be small. This study also had some other weaknesses. Although children were instructed to wear the devices during organized sports programs, children sometimes indicated that they removed the devices because they perceived them as uncomfortable. Although fast developing innovations facilitate application of smaller and thus more comfortable devices combining accelerometry and GPS loggers (e.g., smartphone applications), extensive studies are warranted to validate their performance both in PA and location assessment.

Although we have found some interesting and statistically significant associations that were consistent with previous literature, the direct influence attributes of the objectively measured environment on actual minutes PA or active transport was relatively small in our study. Also, investigating PA behavior in a school setting requires advanced statistical multilevel methodologies. In addition, children’s context-specific PA tends to deviate from normal distributions and non-parametric or transformed models are needed. In our study, log-transformations and standardization of independent variables may have hampered straightforward interpretation of these influences. In addition, based on the relatively low ICC for light PA, modeling random variability of the school-level may not have been absolutely necessary.

This study focused on improving the understanding of relationships between children’s daily physical environment and their afterschool PA behavior. However, according to the socioecological model, the influence of the physical environment interacts with other types of the environment such as parental rules regarding children’s PA, or the social influence of peers regarding PA initiatives. In addition, child characteristics such as attitudes or habits also play a major role in determining children’s afterschool PA participation. With the improved understanding about how attributes of the physical environment influence PA behavior, future studies or policy makers are warranted to implement interventions that contain aspects from multiple types and at different levels of the environment [84].

### 4.4. External Validity

Although we only investigated data within the municipality border from which GIS data was available, findings of this study may be generalizable to other environments with comparable meteorological circumstances, afterschool time segments, population density and residential density. As the Netherlands also have facilities that support cycling (i.e., separate cycling paths), our results may have limited generalizability to environments with less favorable infrastructure for active transport. In addition, findings of this study may be generalizable to samples with comparable distances from children’s residences to their primary schools, and similar motives for active transport. In addition, our sample was considered highly educated; this could imply that child or parental motives regarding leisure time PA may be different from other samples. In our multivariate analyses, we tried to adjust for social economic status using the neighborhood SES-score that was based on relatively small administrative ZIP-code units (average number of the total households in 2016 was 3371 (SD = 1112.10) per area. As in our sample children did not automatically attend the primary school closest to their residence, there was only a small relationship between children attending the same school and their neighborhood SES-score. In addition, time constraints due to competing activities such as organized sports participation or homework may also be considered in comparing results with other studies. However, as we separately investigated cycling, walking and leisure time, results of leisure time PA may be generalizable to less cycle-friendly environments.

## 5. Conclusions

We demonstrated that with combined accelerometer and GPS methodologies, it is possible to investigate associations between the physical environment and context-specific afterschool PA behavior. In addition, by identifying environmental exposure as individual activity spaces that are valid for the specific population and target behavior, enable researchers to better understand how objectively measured features of the built and natural environment plays a role in children’s afterschool PA behavior. We found that greenspaces (i.e., lawns and shrubs) and smaller distances from children’s residences to their school were associated with more afterschool leisure time PA and walking.

## Figures and Tables

**Table 1:** Characteristics of the study population (*n* = 255).

	*n* (%)
Gender; *n* boys (missing *n* = 0)	117 (45.9%)
Age; mean years (SD) (missing *n* = 0)	12.1 (0.5)
Valid measurement-days; *n* ≥ 3 days (missing *n* = 0)	189 (74.1%)
Respondent questionnaire; *n* mothers (missing *n* = 54 (21.2%))	146 (57.3%)
Most frequently used transport mode to school; *n* bicycling (missing *n* = 59 (23.1%))	128 (50.2%)
Most frequently used transport mode to school; *n* walking (missing *n* = 59 (23.1%))	52 (20.4%)
Average daily time spent on homework during measurement; *n* ≤ 10 min (missing *n* = 59 (23.1%))	150 (58.8%)
Standardized mean of social economic status score of the neighbourhood (SD) ^1^ (missing *n* = 0)	0.06 (1.31)

Missing values indicate participants that provided valid GPS and accelerometer data but did not fill in the questionnaire. ^1^ Compared to the National Dutch average, based on the respondent’s postcode area, including the average income per household and the percentages of households with low income, without a paid job and with a lower educational level.

**Table 2:** Afterschool physical activity in contextual domains.

	*n* of Participants		Unadjusted Median (IQR)		*n* of days
		Total Minutes (Including Sedentary)	Minutes in Light PA	Minutes in MVPA	≥80% of Time within Activity Space
Residential parcel †	255	239.3 (138.7)	89.8 (57.4)	8.3 (14.2) ^1^	790 (100%)
School grounds †	233	33.5 (73.3)	15.5 (33.0)	13.5 (35.0) ^2^	640 (100%)
Sports grounds †	214	100.2 (55.2)	54.8 (34.7)	41.0 (29.3) ^2^	202 (42.3%)
Afterschool childcare †	144	19.8 (39.7)	10.0 (21.7)	2.2 (13.2)	181 (67.0%)
Shopping centers †	220	79.5 (105.1)	44.5 (58.5)	8.0 (11.0)	216 (45.6%)
Active transport ‡	253	40.3 (32.7)	23.3 (22.3)	15.0 (17.5)	480 (63.3%)
Passive transport ‡	186	19.5 (14.7)	10.3 (8.2)	1.8 (2.3)	45 (13.8%)
Leisure time *	255	171.2 (136.2)	81.5 (71.5) ^1^	22.3 (24.8)	544 (68.0%)

Children who did not participate in a specific context did not contribute to the calculation of the median minutes. Bold categories highlight the focus of subsequent analyses. ^1^ Contextual domain based on the accumulation of GPS points falling within a 10 m buffer surrounding the polygon parcel(s). ‡ Contextual domain based on the accumulation of GPS points certifying a certain speed threshold. * Afterschool PA and GPS data that remained after the excluding all categories above. Gender differences were investigated using log-transformed multilevel linear mixed models, adjusting for context-specific wear time, age, meteorology, social economic status of the neighborhood, and nested structure of days within children and children within schools. ^1^ Mean significantly lower for boys vs. girls. ^2^ Mean significantly higher for boys vs. girls.

**Table 3:** Multivariate associations between minutes spent in afterschool leisure time physical activity, cycling, walking and first-level geographic information within the activity space.

	Afterschool Leisure Time						Afterschool Active Transport					
	Light PA ^1^			Moderate to Vigorous PA ^2^			Bicycling ^3^			Walking ^4^		
	Unstd. Beta (SE)	Std Beta (SE)	*p*-Value	Unstd. Beta (SE)	Std. Beta (SE)	*p*-Value	Unstd. Beta (SE)	Std. Beta (SE)	*p*-Value	Unstd. Beta (SE)	Std. Beta (SE)	*p*-Value
Wear time (total minutes)	0.01 (000)	0.37 (0.01)	<0.05	<0.00 (0.01)	0.12 (0.01)	<0.05	<0.00 (0.01)	<0.00 (0.01)	0.54	<0.00 (0.01)	0.01 (0.01)	0.60
Gender (boys vs. girls)	−0.03 (0.02)	−0.03 (0.02)	0.09	0.07 (0.03)	0.07 (0.03)	<0.05	−0.02 (0.02)	−0.02 (0.02)	0.46	0.01 (0.03)	0.01 (0.03)	0.76
Age (years)	−0.04 (0.02)	−0.02 (0.01)	<0.05	−0.03 (0.03)	−0.01 (0.01)	0.29	<0.00 (0.02)	<0.00 (0.01)	0.77	−0.02 (0.03)	−0.01 (0.01)	0.57
Temperature (mean degrees Celsius)	<0.00 (0.001)	0.01 (0.01)	0.28	−0.01 (0.002)	−0.04 (0.02)	<0.05	0.01 (0.03)	0.03 (0.01)	<0.05	<0.00 (0.01)	0.03 (0.02)	0.06
Atmospheric pressure (mean hPa)	0.00 (0.001)	0.02 (0.01)	0.07	0.01 (0.002)	<0.00 (0.01)	0.69	<0.00 (0.01)	0.03 (0.01)	0.06	<0.00 (0.01)	0.03 (0.02)	<0.05
Rain (0.2–10.0 mm vs. no rain)	−0.06 (0.02)	−0.06 (0.02)	<0.05	−0.17 (0.06)	−0.17 (0.06)	<0.05	−0.16 (0.06)	−0.16 (0.06)	<0.05	−0.15 (0.07)	−0.15 (0.07)	<0.05
Wind (mean km/h)	<0.00 (0.01)	<0.01 (0.01)	0.91	<0.00 (0.01)	−0.01 (0.01)	0.35	<0.00 (0.01)	<0.00 (0.01)	0.79	<0.00 (0.01)	−0.02 (0.02)	0.23
Solar exposure (UV index)	<0.00 (0.01)	−0.01 (0.01)	0.28	0.01 (0.01)	0.01 (0.01)	0.26	0.01 (0.01)	0.02 (0.01)	0.20	−0.03 (0.01)	−0.04 (0.02)	<0.05
Total area of activity-space (per square km)	0.02 (0.02)	<0.01 (0.01)	0.37	−0.06 (0.03)	−0.03 (0.01)	0.06	−0.08 (0.03)	0.03 (0.01)	<0.05	−0.12 (0.04)	−0.05 (0.01)	<0.05
Roads ^5^	<0.00 (0.01)	0.01 (0.01)	0.35	<0.00 (0.01)	0.03 (0.01)	0.06	<0.00 (0.01)	−0.01 (0.01)	0.56	<0.00 (0.01)	0.04 (0.02)	<0.05
Water ^5^	<0.00 (0.01)	−0.03 (0.01)	0.07	<0.00 (0.01)	<0.00 (0.01)	0.92	<0.00 (0.01)	−0.01 (0.01)	0.38	<0.00 (0.01)	<0.00 (0.01)	0.56
Vegetated terrain ^5^	<0.00 (0.01)	<0.01 (0.01)	0.97	<0.00 (0.01)	<0.00 (0.01)	0.81	<0.00 (0.01)	−0.02 (0.01)	0.09	<0.00 (0.01)	0.03 (0.02)	0.07
Buildings ^5^	<0.00 (0.01)	0.01 (0.01)	0.35	<0.00 (0.01)	<0.00 (0.01)	0.67	−0.02 (0.01)	−0.07 (0.01)	<0.05	0.01 (0.01)	0.03 (0.02)	<0.05

Note: Multivariate linear mixed models, adjusting for context-specific wear time, age, meteorology, social economic status of the neighborhood, and the nested structure of days within children and children within schools. Dependent variables are log-transformed and independent variables were standardized. ^1^ -2 log likelihood = 1383.8; ^2^ -2 log likelihood = 3085.1; ^3^ -2 log likelihood = 1377.5; ^4^ -2 log likelihood = 1711.7; ^5^ Unstandardized coefficients represent the relative area of that variable, as a percentage of a child’s total activity space.

**Table 4:** Multivariate associations between minutes spent in afterschool leisure time physical activity and second-level geographic information within the activity space.

	Leisure Time LPA ^1^			Leisure Time MVPA ^2^		
	Unstd. Beta (SE)	Std. Beta (SE)	*p*-Value	Unstd. Beta (SE)	Std. Beta (SE)	*p*-Value
Total area of activity space (per square km.)	−0.08 (0.03)	−0.03 (0.01)	<0.05	−0.08 (0.03)	−0.03 (0.01)	<0.05
Water ^3^	<0.00 (0.002)	−0.01 (0.01)	0.48	<0.00 (0.004)	<0.00 (0.01)	0.75
Buildings ^3^	<0.00 (0.004)	0.02 (0.01)	0.18	<0.00 (0.002)	0.01 (0.01)	0.60
Vegetated terrain: Agriculture ^3^	0.02 (0.01)	0.02 (0.01)	<0.14	-	-	-
Vegetated terrain: Lawns ^3^	0.03 (0.01)	0.03 (0.01)	<0.05	0.05 (0.01)	0.02 (0.01)	<0.05
Vegetated terrain: Shrubs ^3^	0.63 (0.17)	0.04 (0.01)	<0.05	0.74 (0.21)	0.05 (0.01)	<0.05
Roads: Highway (120 kmph) ^3^	−0.05 (0.02)	−0.03 (0.01)	<0.05	-	-	-
Roads: Local road (50 kmph) ^3^	0.02 (0.01)	0.04 (0.01)	<0.05	-	-	-
Roads: Pedestrian path ^3^	-	-	-	0.02 (0.003)	0.05 (0.01)	<0.05

Multivariate linear mixed models, adjusted for context-specific wear time, age, meteorology, social economic status of the neighborhood, and nested structure of days within children and children within schools. Dependent variables are log-transformed and independent variables were standardized. -: Variable not statistically significant and therefore not part of final multivariate model. ^1^ -2 log likelihood= 516.0; ^2^ -2 log likelihood= 996.5; ^3^ Unstandardized coefficients represent the relative area of that variable, as a percentage of a child’s total activity space.

**Table 5:** Multivariate associations between minutes spent in afterschool active transport and first-level geographic information within the activity space.

	Bicycling ^1^			Walking ^2^		
	Unstd. Beta (SE)	Std. Beta (SE)	*p*-Value	Unstd. Beta (SE)	Std. Beta (SE)	*p*-Value
Total area of activity space (per square km)	0.08 (0.03)	0.03 (0.01)	<0.05	−0.16 (0.04)	−0.07 (0.02)	<0.05
Water ^3^	<0.00 (0.003)	−0.01 (0.01)	0.49	<0.00 (0.001)	<0.00 (0.01)	0.76
Buildings ^3^	−0.02 (0.001)	−0.08 (0.01)	<0.05	0.01 (0.002)	0.02 (0.02)	0.15
Vegetated terrain: Agriculture ^3^	-	-	-	0.01 (0.004)	0.04 (0.02)	<0.05
Vegetated terrain: Lawns ^3^	−0.03 (0.01)	−0.03 (0.01)	<0.05	-	-	-
Vegetated terrain: Shrubs ^3^	-	-	-	0.49 (0.23)	0.03 (0.02)	<0.05
Roads: Main road (100 kmph) ^3^	0.06 (0.02)	0.03 (0.01)	<0.05	0.07 (0.03)	0.04 (0.02)	<0.05
Roads: Pedestrian path ^3^	−0.01 (0.002)	−0.04 (0.01)	<0.05	0.02 (0.005)	0.07 (0.02)	<0.05
Roads: Pedestrian area ^3^	0.23 (0.05)	0.06 (0.01)	<0.05	-	**-**	-

Multivariate linear mixed models, adjusted for context-specific wear time, age, meteorology, social economic status of the neighborhood, and nested structure of days within children and children within schools. Dependent variables are log-transformed and independent variables were standardized. -: Variable not statistically significant and therefore not part of final multivariate model. ^1^ -2 log likelihood= 669.3; ^2^ -2 log likelihood= 992.6; ^3^ Unstandardized coefficients represent the relative area of that variable, as a percentage of a child’s total activity space.

## Appendix A

**Table A1:** Descriptive statistics of all independent variables.

	**Mean (SD)**	**Median (IQR)**
**Child characteristics (*n* = 255 children)**		
Age	12.1 (0.49)	12.06 (0.69)
Gender (n boys)	117 (45.9%)	
**Meteorology (*n* = 808 days)**		
Temperature (mean degrees Celsius)	19.0 (5.4)	17.6 (6.47)
Atmospheric pressure (mean hPa)	1019.0 (6.3)	1018.9 (8.5)
Rain (days with no rain in afterschool period)	538 (66.7%)	
Wind (mean kmph)	8.2 (4.2)	7.1 (4.4)
Solar exposure (UV index)	2.2 (1.3)	2.0 (1.8)
**Afterschool Physical Activity (*n* = 808 days)**		
Leisure Time Light PA per day (minutes)	25.8 (29.4)	16.8 (36.3)
Leisure Time MVPA per day (minutes)	3.9 (8.8)	0.8 (3.7)
Bicycling per day (minutes)	18.8 (0.4)	15.3 (19.8)
Walking per day (minutes)	15.3 (0.4)	10.3 (16.8)
	**Mean (SD)**	**Percent from total area activity space (SD) ***
**Physical environment first level (*n* = 255 children)**		
Total area of activity-space (square kilometres)	1.11 (0.41)	100
Roads (square kilometres)	0.33 (0.14)	29.48 (5.12)
Water (square kilometres)	0.19 (0.21)	15.96 (14.75)
Vegetated terrain (square kilometres)	0.36 (0.26)	32.32 (18.08)
Buildings (square kilometres)	0.02 (0.07)	19.56 (3.79)
**Physical environment second level (*n* = 255 children)**		
Vegetated terrain: agriculture (square meters)	74783.25 (84832.00)	6.70 (7.54)
Vegetated terrain: lawns (square meters)	7557.25 (13044.63)	0.60 (1.05)
Vegetated terrain: shrubs (square meters)	323.70 (1266.03)	0.02 (0.06)
Roads: local road (50 kmph) (square meters)	152007.18 (66805.78)	13.69 (2.37)
Roads: main road (100 kmph) (square meters)	1906.08 (8901.44)	0.11 (0.52)
Roads: highway (120 kmph) (square meters)	2112.11 (5792.11)	0.18 (0.55)
Roads: pedestrian path (square meters)	91586.26 (45885.05)	8.31 (2.98)
Roads: pedestrian area (square meters)	3571.09 (2783.07)	0.33 (0.26)

*: Percent from the individually determined activity space, based on children’s residence, their school and the daily transport route between home and school. In all multivariate models, percentages from total area activity space were used to account for differences between the size of children’s activity space.
